# Major Advances in Emerging Degrader Technologies

**DOI:** 10.3389/fcell.2022.921958

**Published:** 2022-06-22

**Authors:** Hang Luo, Li Wu, Yujian He, Chong Qin, Xinjing Tang

**Affiliations:** ^1^ School of Chemical Sciences, University of Chinese Academy of Sciences, Beijing, China; ^2^ State Key Laboratory of Natural and Biomimetic Drugs, School of Pharmaceutical Sciences, Peking University, Beijing, China; ^3^ School of Medicine and Pharmacy, Ocean University of China, Qingdao, China

**Keywords:** degrader technologies, emerging PROTACs, analogous PROTACs, ubiquitin-proteasome system, lysosomal degradation

## Abstract

Recently, degrader technologies have attracted increasing interest in the academic field and the pharmaceuticals industry. As one of the degrader technologies, proteolysis-targeting chimeras (PROTACs) have emerged as an attractive pharmaceutical development approach due to their catalytic ability to degrade numerous undruggable disease-causing proteins. Despite the remarkable progress, many aspects of traditional PROTACs still remain elusive. Its expansion could lead to PROTACs with new paradigm. Currently, many reviews focused on the design and optimization strategies through summarizing classical PROTACs, application in diseases and prospect of PROTACs. In this review, we categorize various emerging PROTACs ranging from simply modified classical PROTACs to atypical PROTACs such as nucleic acid-based PROTACs, and we put more emphasis on molecular design of PROTACs with different strategies. Furthermore, we summarize alternatives of PROTACs as lysosome-targeting chimeras (LYTACs) and macroautophagy degradation targeting chimeras (MADTACs) based on different degradation mechanism despite of lysosomal pathway. Beyond these protein degraders, targeting RNA degradation with the potential for cancer and virus therapeutics has been discussed. In doing so, we provide our perspective on the potential development or concerns of each degrader technology. Overall, we hope this review will offer a better mechanistic understanding of emerging degraders and prove as useful guide for the development of the coming degrader technologies.

## Introduction

Different from the most of traditional small molecule inhibitors that occupy the active site of protein of interest (POI) (Bedard et al., 2020), degrader technologies hijack natural degradation pathway for targeting protein degradation. For example, PROTACs are heterobifunctional molecules consisting of two ligands, connected via a linker. One ligand enables to target the POI and the other is used for binding to an E3 ligase. PROTACs hijack the ubiquitin-proteasome system (Glickman and Ciechanover, 2002) (UPS) for the POI degradation by forming a POI-PROTAC-E3 ligase ternary complex (Sakamoto et al., 2001). Up to now, various proteins had been successfully degraded, such as androgen and estrogen receptors (AR (Shibata et al., 2018; Han et al., 2019) and ER (Ohoka et al., 2017; Ohoka et al., 2018; Hu et al., 2019)), the bromodomain and extra-terminal (BET) family epigenetic readers (Lu et al., 2015; Zengerle et al., 2015; Raina et al., 2016; Gadd et al., 2017; Qin et al., 2018; Zhou et al., 2018), and the FK506 binding protein 12 (FKBP12) and its fusion proteins (Nabet et al., 2018). Soluble kinases such as cyclin-dependent kinase 9 (Robb et al., 2017; Bian et al., 2018; Olson et al., 2018) (CDK9) and BCR-ABL (Lai et al., 2016; Shimokawa et al., 2017), and receptor tyrosine kinases (Burslem et al., 2018), such as Bruton’s tyrosine kinase (Buhimschi et al., 2018; Huang et al., 2018; Zorba et al., 2018; Krajcovicova et al., 2019; Sun et al., 2019) (BTK), could be best targeted with PROTAC approach. In addition to covering a broad range of targets against a variety of tumor types (Khan et al., 2020), PROTACs also were used to clearing tau related to neurodegenerative diseases (Chu et al., 2016; Lu et al., 2018; Silva et al., 2019; Wang et al., 2021). Furthermore, a number of PROTACs have entered phase 1/2 clinical trials (Xie et al., 2021). Particularly, the Arvinas company released interim clinical data revealing the powerful potential of PROTAC candidates (ARV-110 targeting-AR, used for treating prostate cancer, and ARV-471-an ERα degrader-used for breast cancer therapy), prefiguring the pharmaceutical potential of these PROTAC compounds (Mullard, 2019).

However, the booming PROTACs have inherent shortcomings at the same time, such as off-target toxicity, on-target effect, uncontrollable catalytic degradation, and restricted acting location (Moreau et al., 2020; Garber, 2022). Some of the drug design strategies were exploited to optimize classical PROTACs and construct newer degraders. Light-controllable PROTACs have achieved protein degradation in a spatiotemporal manner. The cancer selective target degradation could be achieved by PROTACs conjugates such as antibody/aptamer/folate-PROTACs. Furthermore, some atypical PROTACs such as covalent PROTACs, trivalent PROTACs and nucleic acid-based PROTACs were explored. In addition to these emerging PROTACs, the degraders via lysosomal pathway such as LYTACs and MADTACs enlarged degradable targets to membrane proteins, extracellular proteins, even non-protein substrates such as intracellular pathogens and dysfunctional organelles. Beyond these protein degraders, several novel bifunctional chimeras aimed to harness catalytical nuclease to direct the RNA target for targeting RNA degradation, which would expand the drug development toolboxes from cancer to viruses.

The blowout development of PROTACs brought with it a number of impressive reviews, which provided discussions on the design and optimization through summarizing classical PROTACs (Paiva and Crews, 2019; Yang et al., 2019; Wang et al., 2020; He S. et al., 2021; Bemis et al., 2021), application in diseases (Khan et al., 2020; Vogelmann et al., 2020; Dale et al., 2021), prospect (Bond and Crews, 2021; Farnaby et al., 2021) of PROTACs, and degraders via lysosomal pathway (Ding et al., 2020; Alabi and Crews, 2021). Particularly, the PROTACs-derived strategies and other alternatives to realize protein degradation via proteasome or lysosome have just been reviewed (Zhong et al., 2022). Nevertheless, this review mainly discussed the emerging PROTACs and analogues of PROTACs reported since 2019, focusing on their structural characteristics and action modes to improve the mechanistic understanding of these emerging degrader technologies. Moreover, this review categorized various emerging PROTACs and analogous PROTACs technologies based on different perspectives, and analyzed the merits and potential challenges.

## Novel PROTACs Targeted Protein Degradation via UPS

### Light-Controllable PROTACs

Although PROTACs exhibit a superior catalytic behavior for protein degradation and several PROTACs candidates demonstrate powerful potential in clinical trial, the off-target effects remain a challenge. Conditional control can tightly regulate the PROTAC activity, and thus lead to reduced off-target effects, an improved anti-tumor activity and a highly targetable pathway (Nalawansha and Crews, 2020). Light is applied in various biological systems with spatiotemporal control due to its noninvasive nature. Several strategies with two kinds of photoactive ligands (caging and photoswitching groups) have been established to control PROTAC activity using light.

**FIGURE 1:**
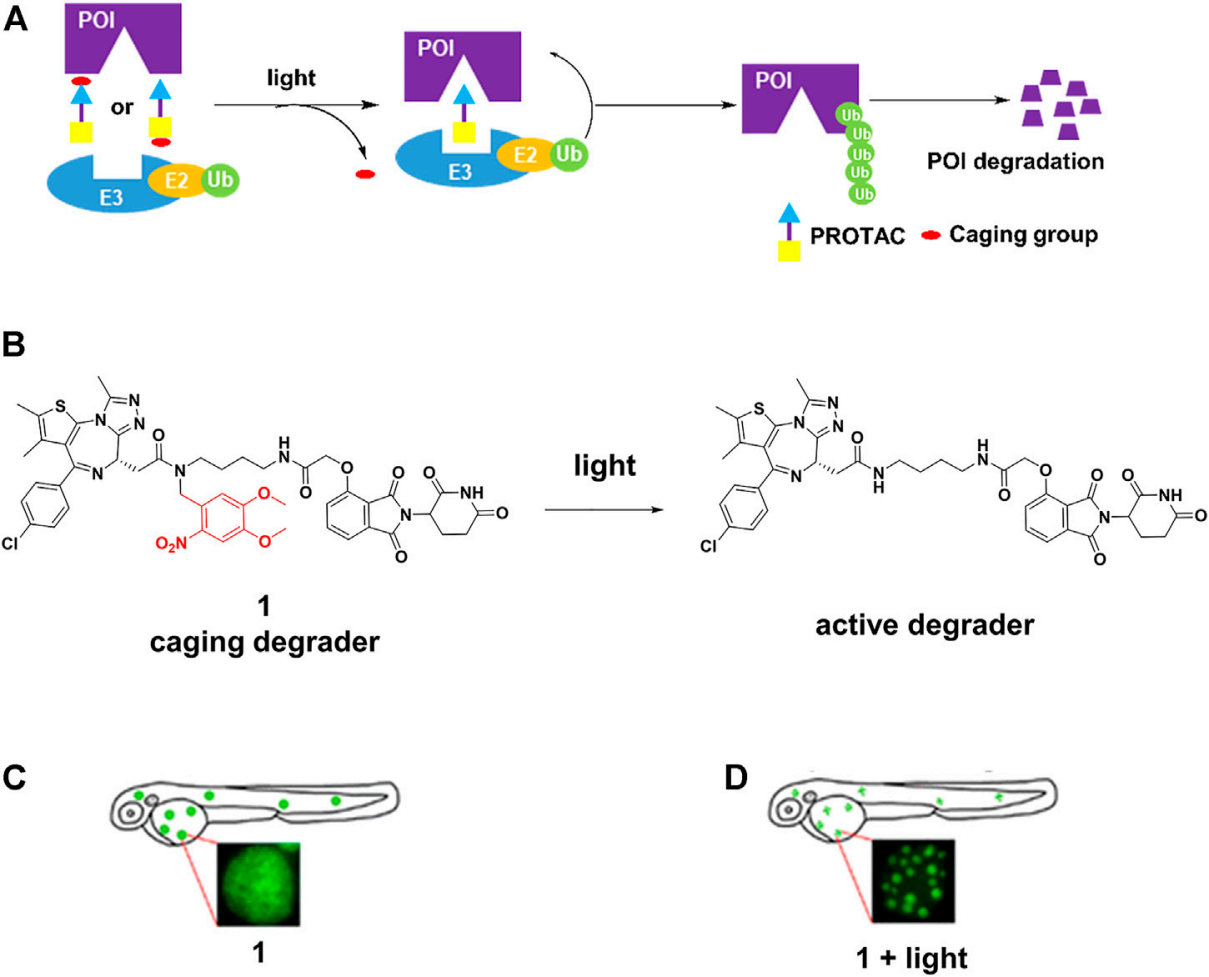
**(A)** Action mode of caging PROTACs. **(B)** Uncaging reaction of caging degrader **1**. **(C)** Degradation of BRD4-EGFP fusion protein through **1** without light irradiation in zebrafish embryos (Adapted with permission from (Xue et al., 2019). Copyright (2019) American Chemical Society). **(D)** Degradation of BRD4-EGFP fusion protein through **1** with light irradiation in zebrafish embryos (Adapted with permission from (Xue et al., 2019). Copyright (2019) American Chemical Society.).

The caging strategy is based on the inactivity of PROTACs with a photocleavable group that blocks binding with either the POI or the E3 ligase, uncaging with light at an appropriate wavelength enables the formation of active PROTACs, leading to POI degradation (Figure 1A). Based on the BRD4-JQ1 crystal structures, Pan and others (Xue et al., 2019) presented **1** (Figure 1B) by installing the bulky 4,5-dimethoxy-2-nitrobenzyl (DMNB) group on the crucial chemical group of original PROTAC. **1** was inactive when DMNB blocked inhibitor binding to POI. Upon irradiation with ultraviolet (UV) light, DMNB was removed (Figure 1B), which activated the PROTAC-induced POI degradation in zebrafish embryos (Figure 1C,D). However, it must be kept in mind that CRBN-based PROTACs caged in this way can still function as molecular glues and recruit other substrates to the E3 ligase.

**FIGURE 2:**
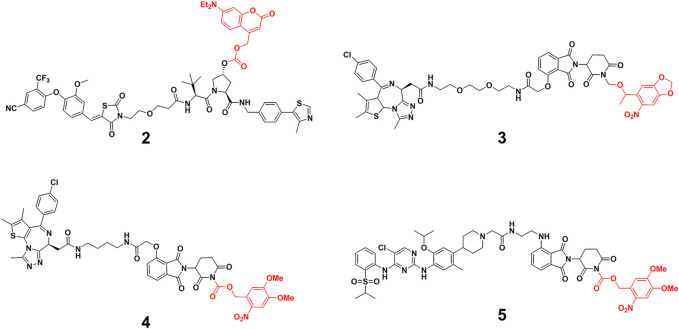
Chemical structure of caging PROTACs.

Caging E3 ligase might mitigate this problem. Deiters and others (Naro et al., 2020) successfully developed diethylamino coumarin (DEACM)-caged estrogen-related receptor α (ERRα) PROTAC **2** and 6-nitropiperonyloxymethy (NPOM)-caged BRD4 PROTAC **3** (Figure 2) to optically control protein degradation by installation of DEACM onto the essential hydroxyl group of von Hippel-Lindau (VHL) ligands or NPOM onto the glutarimide nitrogen of CRBN ligands. They respectively demonstrated robust photoactivation of ERRα and BRD4 degradation in cells at micromolar upon irradiation with light ≤405 nm for 3 min. Wei and others (Liu et al., 2020) prepared Opto-dBET1 (**4**) and Opto-dALK (**5**) (Figure 2) through covalent attachment of a nitroveratryloxy carbonyl group onto the glutarimide nitrogen of pomalidomide to prevent the recruitment of ubiquitin to the CRBN E3 ligase, the restricted BRDs and anaplastic lymphoma kinase (ALK) degradation could be triggered at a specific rate or time upon UV irradiation. Tate and others (Kounde et al., 2020) demonstrated conditional control of BRD4 degradation via the direct installation of the DMNB group onto the essential hydroxyl group of VHL E3 ligands, enabling activation of the caged degrader in the cell with short-term UV irradiation time (60 s).

**FIGURE 3:**
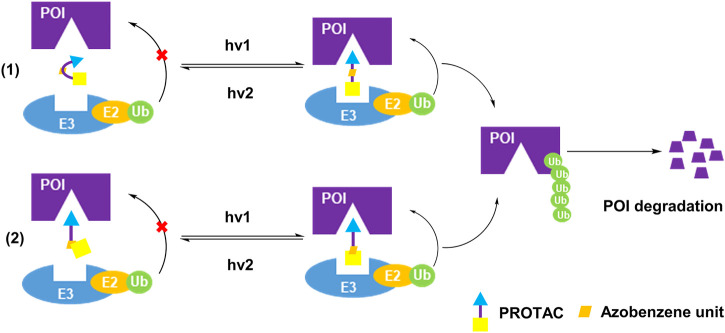
Two action modes of photoswitching PROTAC strategy.

Although the caging strategy enables the degradation of POI in a spatiotemporal manner, it only turns on the activity, and possibly generates some unwanted by-products. Azobenzene is known as one of the smallest photoswitches. It does not substantially increase molecular weight of pharmaceuticals with incorporation of azobenzene, and is widely applied in targeted cancer therapies (Welleman et al., 2020). On the one hand, azobenzene unit became the part of PROTAC linker for controlling the distance between two protein warheads. On the other hand, azobenzene unit was incorporated into the E3 ligand for controlling the binding between E3 ligase and its ligand. In both ways, azobenzene was installed into PROTACs to achieve reversible light-control of PROTAC activity (Figure 3).

**FIGURE 4:**
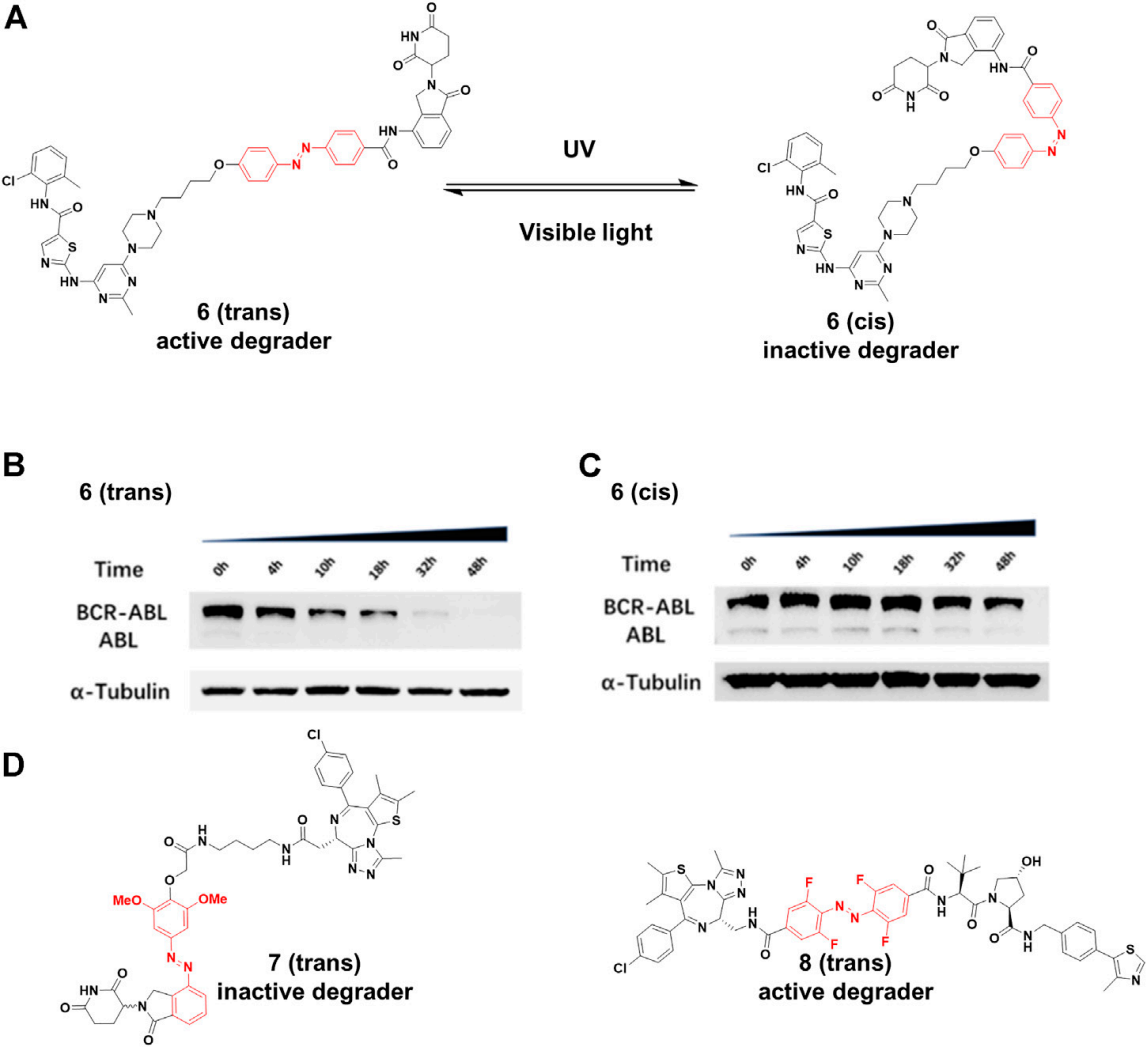
**(A)** Reversible reaction of **6** upon visible light or UV irradiation. **(B)** and **(C)** The time-course Western blot assays of *trans* and *cis-* **6** at 250 nM, respectively (Adapted with permission from (Jin et al., 2020). Copyright (2020) American Chemical Society). **(D)** Chemical structure of photoswitching PROTACs.

Jiang and co-workers (Jin et al., 2020) constructed **6** by installing an azobenzene at the amino in lenalidomide for transforming the PROTAC configuration (Figure 4A), and only *trans*-**6** was active due to the steric hindrance of *cis*-configuration. The time-course analysis showed that the BCR-ABL protein level clearly decreased after 4 h treatment, and more than 90% of BCR-ABL degradation was found for the *trans*-configuration after 32 h treatment (Figure 4B). On the contrary, no noticeable reduction in BCR-ABL was observed for the *cis*-**6** until 32 h treatment (Figure 4C). Trauner and co-workers (Reynders et al., 2020) created **7** (Figure 4D) by incorporating azobenzene into CRBN ligands. Molecular modeling suggested that the *trans* configuration of the **7** could not be bound with CRBN as well as the *cis* configuration. Their results showed that *trans*-**7** had little or no degradation in the dark, but could transform to *cis*-**7** that further degraded BRD2-4 with blue-violet light (380–440 nm). However, without light exposure, *cis*-**7** could slowly isomerize back to the *trans*-configuration in dimethyl sulfoxide at 37°C. Therefore, pulsed irradiation was required to keep the *cis* form for maintaining degradation activity. The half-life of this process can be tailored by chemical substitution. Crews and Carreira group (Pfaff et al., 2019) designed **8** (Figure 4D) by inserting an *ortho*-F_4_-azobenzene linker between both warhead ligands to degrade BRD2. The *trans*-**8** was active because it engaged both protein partners to generate an available ternary complex for degradation. On the contrary, the *cis*-isomer was inactive because the distance defined by the linker was too short (8Å) to form complex between its binding protein partners. Importantly, the azo-*cis*-isomer had long thermal *τ*_*1/2*_ (Knie et al., 2014), while the inactive state of the **8** was persistent, with no requirement for pulsed irradiation.

Light-controllable PROTACs avoid some toxic effects temporally and spatially, making them useful tools in cell biology and as potential options for precision medicine (Reynders and Trauner, 2021). However, most light-controllable PROTACs need UV/visible light for activation, which may cause light-induced cell toxicity. Furthermore, it is difficult for UV/visible light to penetrate into deep tissues. Therefore, future work should focus on activation with near-infrared/infrared light in the promising field of light-controllable PROTACs.

### Antibody/Aptamer/Folate-PROTAC Conjugates

**FIGURE 5:**
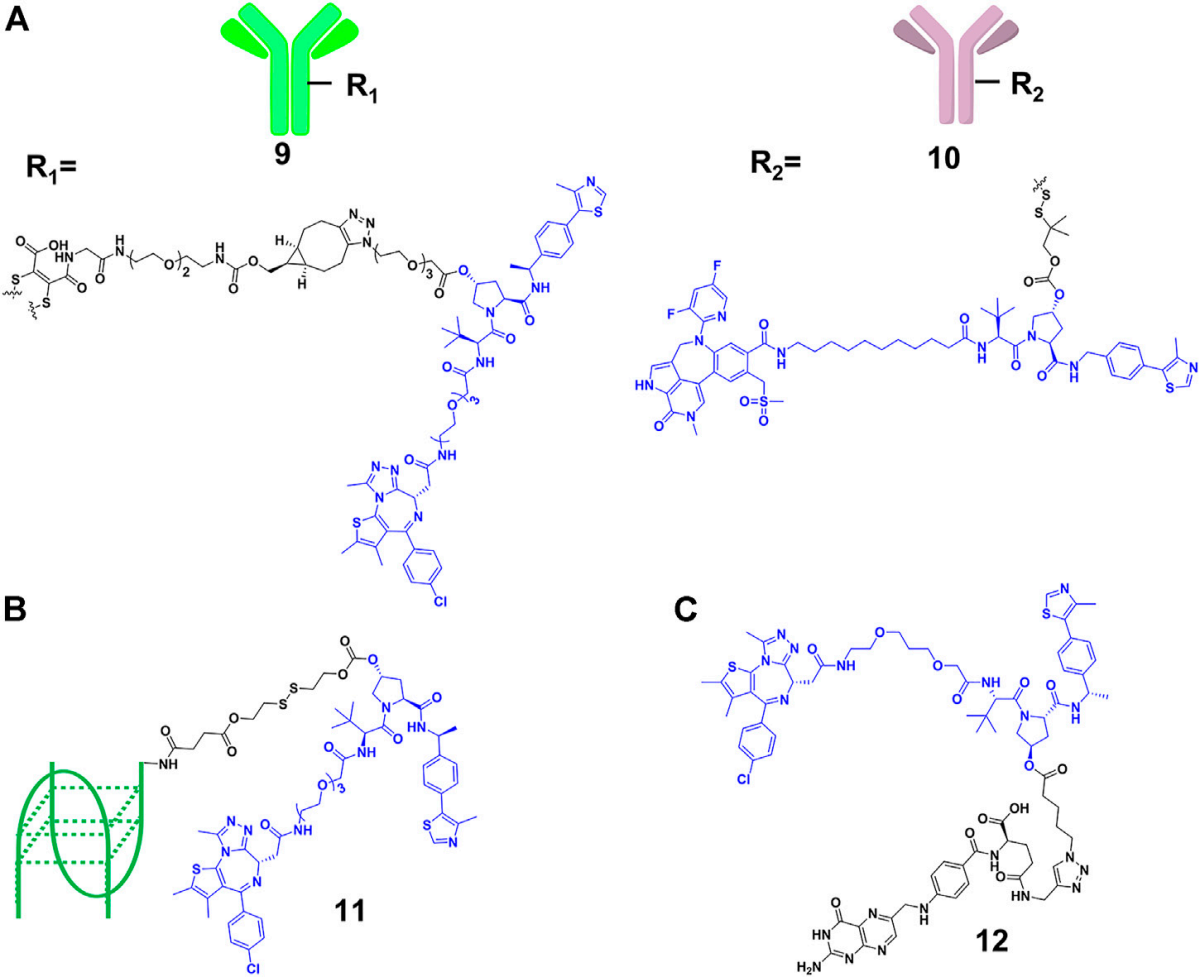
**(A)** Structure of antibody-PROTAC Conjugates. **(B)** Structure of aptamer-PROTAC Conjugates. **(C)** Structure of folate-PROTAC Conjugates.

Although the efficiency of PROTACs have been established for a wide variety of targets, most of the reported PROTACs showed limited tissue selectivity and failed to distinguish different cell types (Liu et al., 2021b). Based on the antibody-drug conjugates (ADCs) strategy (Casi and Neri, 2012), Tate and co-workers (Maneiro et al., 2020) designed **9** (Figure 5A), which is a new example of antibody-PROTACs conjugates (Ab-PROTACs). Because of the antibody-antigen interaction [trastuzumab and human epidermal growth factor receptor 2 (HER2)], **9** selectively targeted BRD4 for degradation in HER2-positive cells, while no degradation effect was found in HER2-negative cells. Dragovich and co-workers (Pillow et al., 2019) constructed **10** (Figure 5A) using a disulfide linker, which selectively targeted the acute myeloid leukemia (AML) tumors by C-type lectin-like molecule-1 (CLL1) antigen-dependent delivery. Moreover, they discovered that **10** had a prolonged *in vivo* exposure, favorable pharmacokinetic properties, and stronger antitumor activity.

However, antibody-PROTACs conjugates were usually not uniform, and their high molecular weight might result in disadvantages such as low uptake efficiency, short plasma half-life and high immunogenicity. Aptamer is also called “chemical antibody”, and is easily synthesized and modified. Moreover, it exhibits low immunogenicity and favorable water solubility, and is widely used in targeted therapies against tumors (Xuan et al., 2018). Sheng’s group (He M. et al., 2021) developed aptamer-PROTAC conjugates **11** (Figure 5B) by connecting the PROTAC for BRD4 degradation to aptamer AS1411 for targeting cancer cells with a cleavable linker. Their results showed that **11** exhibited similar BRD4 degradation efficiency (DC_50_ = 22 nM) comparing with original PROTAC in nucleolin-overexpressed MCF-7 cells, while it did not degrade BRD4 in the control group. More importantly, **11** had better target capabilities than original PROTAC *in vivo*, resulting in enhanced BET degradation, antitumor potency, and low toxicity.

Besides using antibody as the transport carrier, We’s group (Liu et al., 2021b) designed folate-PROTACs **12** (Figure 5C) by installing folate onto with the OH group of a VHL-based PROTAC via an ester bond. Folate receptor α is overexpressed in numerous cancer cells, with very low or no expression in normal cells or tissues (Scaranti et al., 2020). Therefore, folate-PROTACs **12** were preferentially transported into cancer cells through interaction of folate and folate receptor α (FOLR 1), and then cleaved by intracellular hydrolases to release active PROTACs for degradation. Using this delivery strategy, they validated three folate-PROTACs that effectively degraded fusion proteins of BRDs, MEK1/2 and ALK only in cancer cells. But the attachment of folate moiety increased the molecular weight of PROTACs, compromising its oral bioavailability and pharmacokinetics. In addition, further studies will focus on the stability and delivery efficiency of folate-PROTACs *in vivo*.

### Covalent PROTACs

**FIGURE 6:**
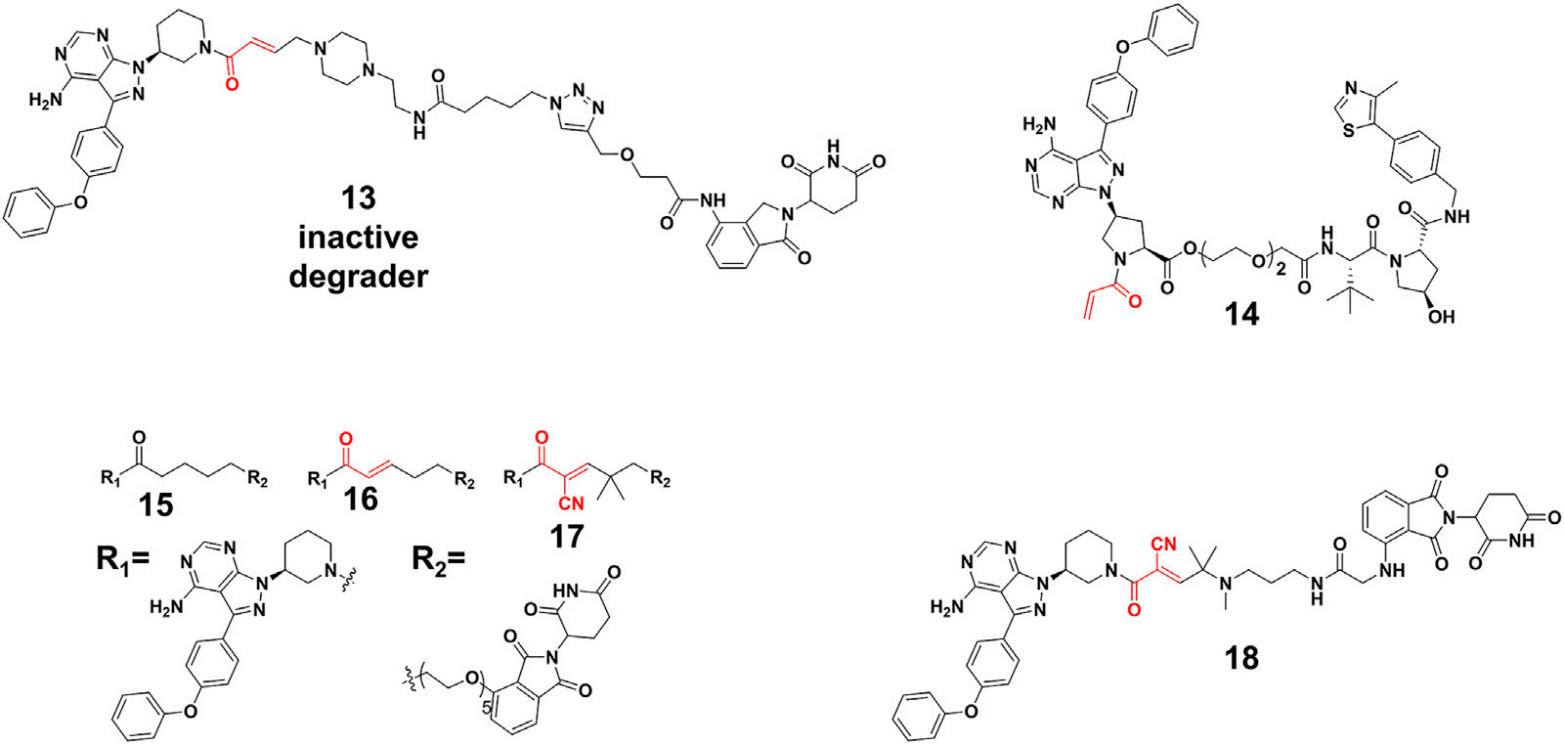
Chemical structures of irreversible covalent, noncovalent, and reversible covalent PROTACs.

Efficient degradation usually requires high affinity between PROTACs and targets. However, it is difficult to acquire high affinity ligand for many intractable targets, such as transcription factors (Koehler, 2010) (TFs) and challenging enzyme classes (Gray et al., 2020). Covalent PROTACs incorporate an electrophile moiety for binding covalently targets, which might be used for the degradation of the aforementioned targets. Harling’s group (Tinworth et al., 2019) prepared a covalent PROTAC **13** (Figure 6) derived from BTK inhibitor ibrutinib, connected with the CRBN E3 ligase ligand. Unfortunately, Western blot and proteomics analyses determined that PROTAC **13** didn’t result in efficient degradation of BTK. Moreover, they proved that the reasons for inactivity were not abnormal engagement of BTK or E3 ligase, linker length and E3 ligase type. This inactivity might also result from issues such as low stability, permeability and unfavorable geometry. For example, **13** harbored a piperazine moiety in the linker, attached one carbon away from the acrylamide group, which might influence acrylamide reactivity and the binding ability of PROTACs.

Interestingly, some covalent PROTACs successfully degraded the POIs. For example, Pan et al. (Xue et al., 2020) developed a covalent PROTAC **14** (Figure 6) based inhibitor ibrutinib for BTK degradation. Different from covalent PROTAC **13**, the carboxyl group on ibrutinib was selected as the egress point to the linker in PROTAC **14**, which might improve reactivity between acrylamide and sulfydryl from BTK because of reduced steric hindrance. With the optimization of the linker and the E3 ligase, PROTAC **14** resulted in an effective degradation for the BTK with the DC_50_ (the concentration causing 50% protein degradation relative to the control group) of 136 nM, as well as the B lymphocyte kinase (BLK) with a DC_50_ of 220 nM. Their results proved that the robust binding potencies of covalent PROTACs appeared to compensate well for the loss of catalytic characteristic, and did not prevent the formation of effective PROTACs. Furthermore, the London’s group (Gabizon et al., 2020) constructed and compared three types of PROTACs. Their study revealed reversible covalent PROTACs **17** (Figure 6) had high potency and selectivity for BTK degradation (DC_50_ = 6 nM), which combined the advantages encompassed by covalent binding, such as increased potency and selectivity, while maintaining the reversibility that was considered significant for the catalytic characteristic of PROTACs. More importantly, they demonstrated the role of covalent binding in the process of degradation. In the time scale of degradation, they discovered that **17** was more efficient than irreversible covalent PROTACs **16** (Figure 6) for covalent bond formation. This indicated that part of degradation by **16** (DC_50_ = 1.9 nM) might derive from reversible binding, but **17** driven degradation was primarily done via covalent engagement. Of note, reversible non-covalent PROTACs **15** (DC_50_ = 2.2 nM) (Figure 6) was a more potent degrader than **17**, which might be the result of the more stable BTK-PROTACs-E3 ligase complex and more degradation recycle times because of rapid binding and dissociation equilibrium. Wang’s group (Guo et al., 2020) applied reversible covalent inhibitors to develop efficient PROTACs **18** (DC_50_ = 6.6 nM) (Figure 6), which ranked high among those of other BTK degraders in target engagement and cell proliferation assays. Importantly, they demonstrated that the reversible covalent cyano-acrylamide unit could lead to the increase of accumulation of PROTACs **18** in cells, which might result from the fast and reversible reactions between the cyano-acrylamide unit and intracellular glutathione (typically in the 1-10 mM range). Additionally, cyano-acrylamide moiety could reversibly react with many available free cysteine residues on the cellular surface, thereby mediating enhanced uptake of the compounds.

**FIGURE 7:**
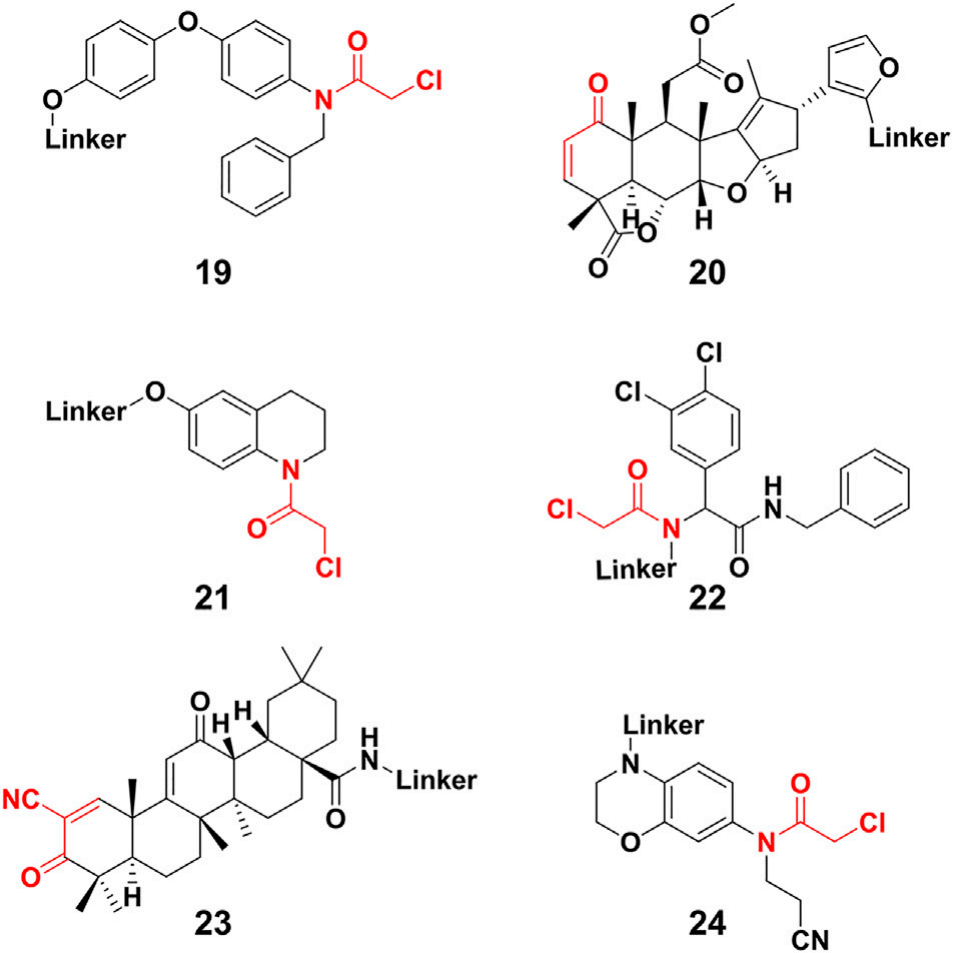
Chemical structure of novel E3 ligase ligand for covalent PROTACs.

Except for the aforementioned covalent PROTACs which covalently engage the POI, there are some degraders that covalently targeting novel E3 ligases for protein degradation (Gabizon and London, 2021). Nomura’s group (Ward et al., 2019) identified a potential RNF4 E3 ligase ligand **19** (Figure 7) through activity-based protein profiling (ABPP)-based covalent ligand screen *in vitro*. Compound **19** (Figure 7) could covalently bind RNF4 by reacting with zinc-coordinating cysteines in the RING domain, with no effect on RNF4 activity. Then the compound **19** was conjugated with BRD4 ligand JQ1 for constructing a degrader, which degraded BRD4 in a proteasome- and RNF4-dependent manner. Nomura’s group (Spradlin et al., 2019) also demonstrated that the anti-cancer natural product nimbolide (**20**, Figure 7) could be a covalent binder for recruiting RNF114 E3 ligase through ABPP platforms. They further transformed nimbolide to a potential BRD4 degrader. Zhang et al. (Zhang et al., 2019; Zhang X. et al., 2021) designed electrophilic PROTACs consisting of the broadly reactive electrophilic fragments and the selective protein ligand for protein degradation. After observing POI degradation, they identified the E3 ligase bound by the electrophilic ligand. Using this screening strategy, they identified respectively DCAF16 (**21**) and DCAF11 (**22**) E3 ligases ligand (Figure 7). Comparing with irreversible covalent ligand, reversible covalent E3 covalent ligand avoided permanent E3 ligase modification. Based the reversible interactions between bardoxolone (**23**) (Figure 7) and cysteines on the KEAP1 E3 ligase, Tong et al. (Tong et al., 2020) constructed degrader through conjugating bardoxolone and JQ1 for BRD4 degradation. Their results showed potent BRD4 degradation depended on the covalent binding to KEAP1. Very recently, Henning et al. (Henning et al., 2022) identified the covalent ligand **24** (Figure 7) that targeting FEM1B E3 ligase, and they transformed compound **24** to potential BRD4 and BCR-ABL degraders respectively. So far, only few E3 ligase was used for constructing PROTACs. Covalent E3 ligase ligand provided the strategy for exploiting new E3 ligase and expanded the toolbox of PROTACs.

### Trivalent PROTACs

**FIGURE 8:**
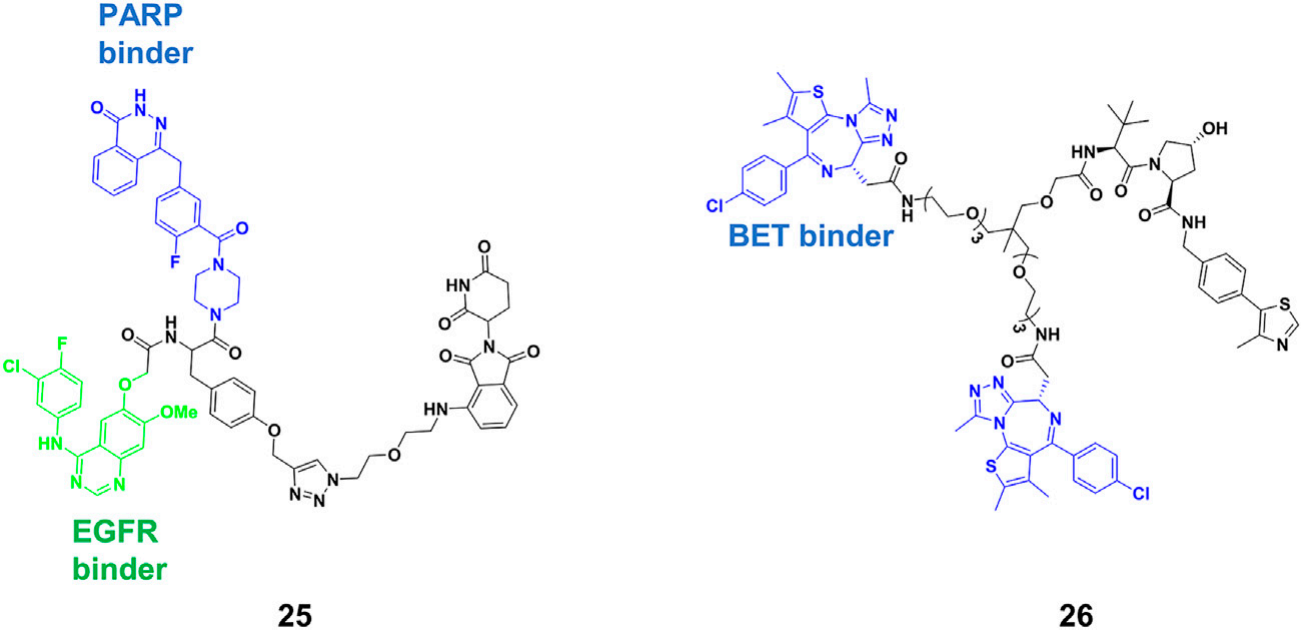
Chemical structure of trivalent PROTACs.

Bispecific antibodies could bind two proteins simultaneously, co-localizing them, and achieved considerable success in treating complex diseases in the past (Brinkmann and Kontermann, 2017). Inspired by bispecific antibodies, Li’s group (Zheng et al., 2021) designed the trivalent PROTACs containing three warheads, in which an E3 ligand interconnect with two different inhibitors by using the tyrosine or serine as a star-type linker. They synthesized a range of trivalent PROTACs based on the CRBN or VHL E3 ligase, and observed that the compound **25** (Figure 8) exhibited degradation ability for both epidermal growth factor receptor (EGFR) and poly (ADP-ribose) polymerase (PARP) at micromolar. One of the reasons of modest degradation efficiency might drive from forming little productive ternary complex. Furthermore, increasing molecular weight of degraders would be accompanied by lack of cellular permeability or poor PKs. In Li’s study, three warheads have different binding targets. It is difficult to produce simultaneously strong binding between all warheads and targets. However, increasing binding valency for the same target is a useful strategy for improving degradation efficiency. Ciulli’s group (Imaide et al., 2021) designed trivalent PROTACs consisting of a bivalent BET inhibitor and VHL ligand tethered via a branched linker such as **26** (Figure 8), in which bivalent BET inhibitor engaged BD2 and BD1 bromodomains to form a 1:1:1 ternary complex with VHL and BET. Their results showed that **26** enhanced binding affinity for BET proteins due to intramolecular BD1 and BD2 binding, and cooperativity within the ternary complex. Further results showed that BRD2-**26**-VHL showed the most favorable ternary complex formation, prolonged residence time and the most robust level of ubiquitination. **26** as a very potent BRD2 degrader (DC_50_ = 1.1 nM) showed more potent anticancer activity compared to bivalent PROTACs. Interestingly, the PK data of **26** suggest that it will be appropriate for *in vivo* use. The trivalent PROTACs need ingenious design and complex chemical synthesis, but it expanded the structural diversity of PROTACs, greatly broadening the application spectrum of the PROTACs strategy.

### Nucleic Acid-Based PROTACs

**FIGURE 9:**
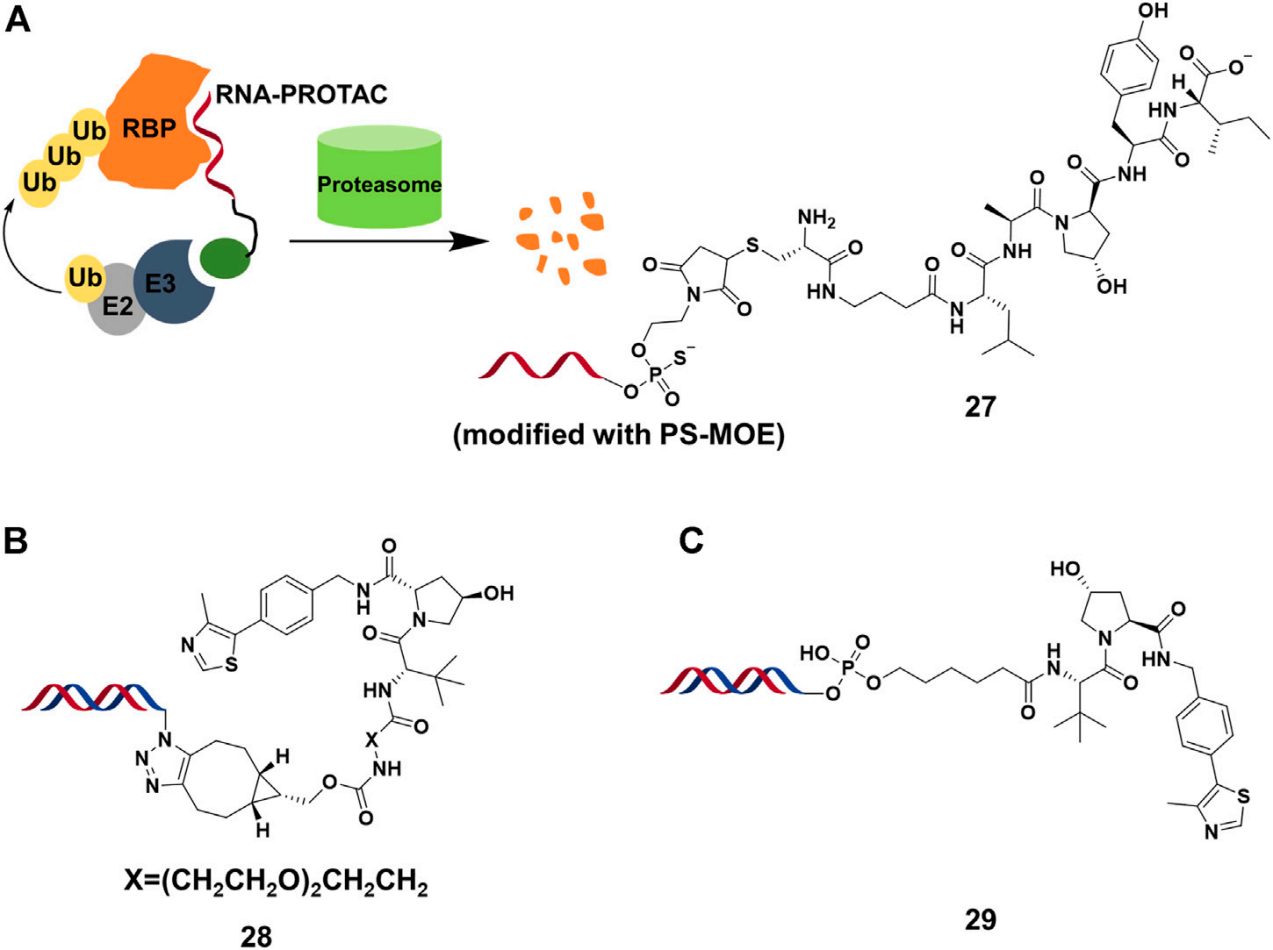
**(A)** The action mode and structure of RNA-PROTACs. **(B)** Structure of TF-PROTACs. **(C)** Structure of oligonucleotide-based PROTACs (O’PROTACs).

RNA binding proteins (RBPs) constitutes a large part of cell’s proteomes, and play important roles in RNA-dependent processes (Gerstberger et al., 2014). Hall’s group (Ghidini et al., 2021) presented the RNA-PROTACs containing oligoribonucleotide and E3-recruting peptide for the degradation of RBPs (Figure 9A). They used oligoribonucleotide 5′-AGGAGAU-3′ to target the Lin28 protein, which was characterized as a potential drug target considering that overexpression of Lin28 promotes the tumor cell proliferation. They used phosphorothioate backbone and modification of ribose 2′-O-methoxyethyl group (PS-MOE) to avoid the cleavage of RNA-PROTAC from ubiquitous nucleases *in vivo*, which showed relatively strong interactions between these modified oligoribonucleotides and the target. Finally, they synthesized RNA-PROTACs **27** (Figure 9A) by conjugating a VHL-recruiting peptide to 5′-end of oligoribonucleotides, and found that **27** had the ability to mediate the ubiquitination of Lin28A and degradation by approximately 50% at 2 μM. Furthermore, this strategy was successfully applied for degrading a splicing factor RNA binding fox-1 homolog (RBFOX1).

A similar strategy was used in the degradation of TFs, which were engaged in genetic regulation by recognizing the specific DNA sequence. It was difficult for small molecule inhibitors to target TFs because it lacked active sites or allosteric regulatory pockets ubiquitously distributed in kinases or others. Wei’s group (Liu et al., 2021a) reported that TF-PROTACs **28** (Figure 9B), which were DNA oligonucleotides that linked to VHL E3 ligase ligand via a linker. They used two unmodified DNA oligonucleotides to target the nuclear factor kappa-light-chain-enhancer of activated B cells (NF-κB) and E2F1 transcription factor, and optimized the length and structure of the linker. The result showed that p65 was degraded almost completely by **28** at 10 μg/ml, and displayed superior antiproliferative effects in cells. Recently, Huang’s group (Shao et al., 2021) reported a similar study in which they demonstrated that highly cancer-related TF, lymphoid enhancer-binding factor 1 (LEF1), was efficiently degraded by O’PROTACs **29** (DC_50_ = 25 nM) (Figure 9C). More importantly, O’PROTACs inhibited TF’s transcriptional activity and suppressed tumor cell proliferation *in vivo*. These studies illustrated that nucleic acid could serve as a POI binder in PROTACs, and expanded the degradation spectrum of PROTACs. Further studies might focus on the optimizing structure of nucleic acid binder for improving binding ability and stability, as well as the cell permeability of nucleic acid-based PROTACs.

## Hijacking Non-UPS Pathway for Protein Degradation

### Lysosomal-Targeting Chimeras (LYTACs)

Extracellular POIs are inaccessible to PROTACs due to the intracellular existence of the UPS, but the lysosomal pathway is an alternative approach and not restricted to POIs encompassing intracellular domains (Ding et al., 2020). Recently, a series of lysosomal-targeting chimeras were developed to clear extracellular and transmembrane POIs.

**FIGURE 10:**
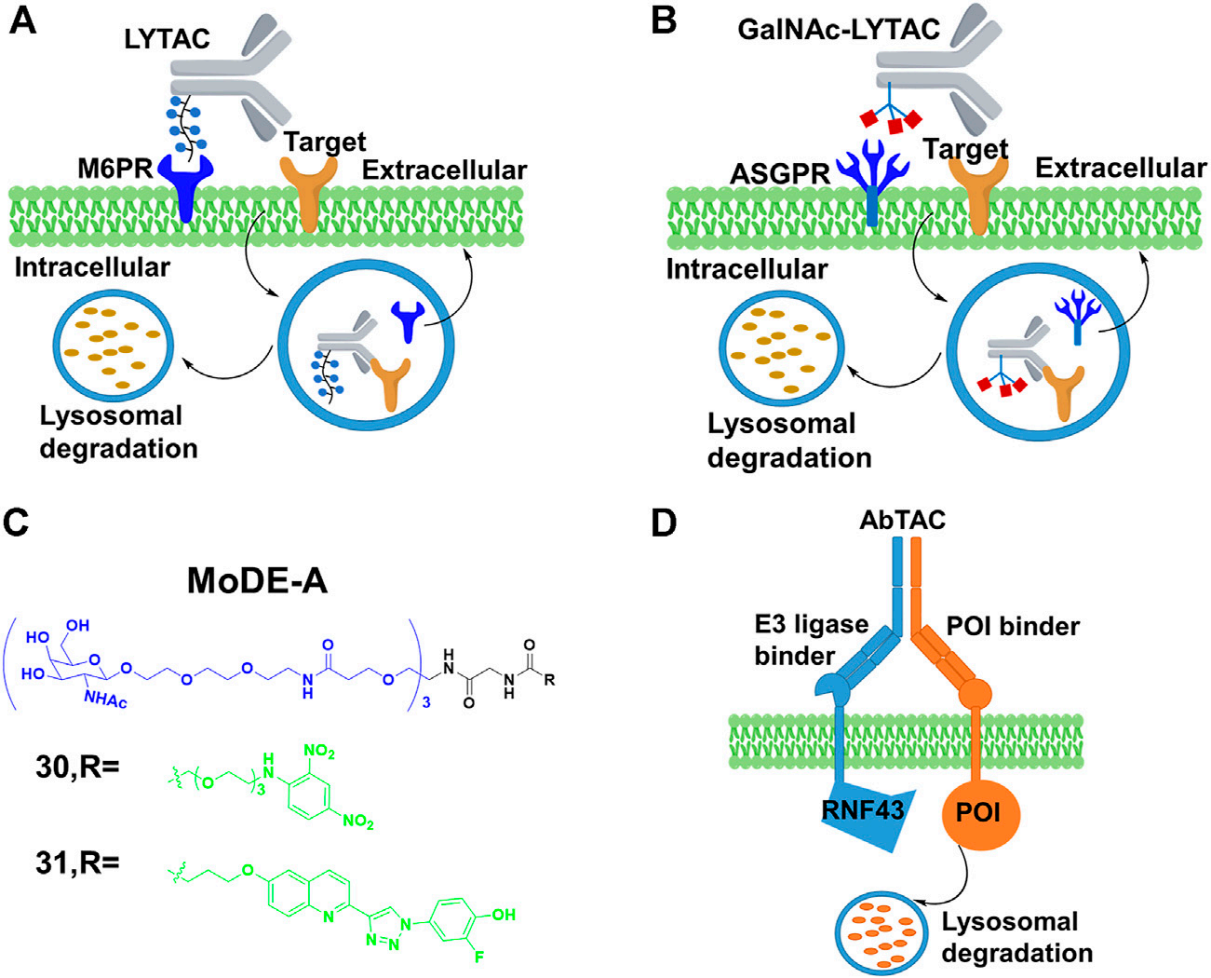
**(A)** Action mode of LYTAC. **(B)** Action mode of N-acetylgalactosamine (GalNAc)-LYTAC. **(C)** Chemical structure of molecular degrader of extracellular proteins through the Asialoglycoprotein receptor (MoDE-A). **(D)** Action mode of antibody-based PROTAC (AbTAC).

Cation-independent mannose-6-phosphate receptor (CI-M6PR), as one of lysosome-targeting receptors (LTRs) on the cell surface, binds to the mannose 6-phosphate (M6P)-containing POI. Also, it transports POI to lysosomes, where acidic pH induces the release of the glycosylated POI for degradation while CI-M6PR is recycled (Coutinho et al., 2012). Based on this special degradation pathway, the Bertozzi group (Banik et al., 2020) constructed LYTACs by conjugating the synthetic glycopolypeptide ligands M6Pn to serine or lysine residues on the antibody, in which the antibody was used to bind POI, and then the POI with the M6Pn tag was transported to lysosome for degradation (Figure 10A). For example, the EGFR monoclonal antibody cetuximab was connected to M6Pn, resulting in 70% degradation of EGFR in cells. The maximal degradation degree needed treatment time between 12 and 24 h, and the started degradation point occurred after 3 h. Furthermore, they also achieved the degradation of CD71 (transferrin receptor 1) and PDL1 (Programmed cell death one ligand 1) to demonstrate the scope of the LYTAC platform. Based on the aforementioned study, Bertozzi group (Ahn et al., 2021) engaged the liver-specific asialoglycoprotein receptor (ASGPR) for constructing the cell-type-specific LYTACs. They constructed GalNAc-LYTACs composed of an antibody against the POI conjugated to homogeneous GalNAc ligands for degradation (Figure 10B). ASGPR recognized glycoproteins with GalNAc ligands and internalized them by inducing receptor-mediated endocytosis. Following internalization and endosomal acidification, GalNAc was released and ASGPR recycled to the surface of the plasma membrane, while the glycoproteins proceeded to the lysosome (Stockert, 1995). The results of Bertozzi group showed that the EGFR and HER2 could be degraded by GalNAc-LYTACs in hepatocellular carcinoma (HCC) cells. In addition, they constructed the GalNAc-LYTAC based on a synthetic peptide conjugating to a tri-GalNAc (triantennary N-acetylgalactosamine). To be excited, this GalNAc-LYTAC bearing a peptide enabled successful degradation of integrins and resulted in excellent antiproliferative effects. In addition, they observed that the variations in antibody conjugation sites and GalNAc-to-antibody ratios influenced degradation efficiency and pharmacokinetic profiles *in vivo*. Similarly, Tang’s group (Zhou et al., 2021) created a series of degraders by using tri-GalNAc to conjugate biotin, antibodies, or segments of antibodies. They observed the mouse IgG-647 and EGFR degradation in varying degree. In addition, they prepared some degraders with different molecular weights, and demonstrated that the small degrader-antibody complexes were facilitated to internalization relative to the big complexes. Different from the aforementioned two studies, Spiegel’s group (Caianiello et al., 2021) designed a class of bifunctional small molecules termed MoDE-A, which was composed of trivalent GalNAc, a PEG linker and a small-molecule ligand for binding the POI. This chimera was relatively small size and easily synthesized compared with LYTACs. The authors showed that **30** and **31** (Figure 10C) could successfully trigger the degradation of antibody and extracellular protein *in vivo* respectively.

In addition, Well’s group (Cotton et al., 2021) designed the bispecific antibody that recruited the E3 ligase for PDL1 degradation, which was called AbTAC (Figure 10D). Ring finger protein 43 (RNF43) was a single-pass E3 ligase comprising the extracellular and intracellular RING domain that was convenient to generate antibodies by phage display. Therefore, they prepared a bispecific antibody AC-1, with its one arm binding RNF43 and the other binding the cell-surface immune checkpoint protein PDL1. Their results showed that AC-1 had high degradation efficiency with a DC_50_ = 3.4 nM after 24 h in MDA-MB-231 cells. Furthermore, the PDL1 degradation in other cancer cells was also observed. Han’s group (Miao et al., 2021) developed bispecific aptamer conjugates which bound both the IGFIIR (lysosome-shuttling receptor on the cellular surface) and the POI on the cellular membranes, and then shuttled the POI to the lysosomes for degradation. They demonstrated that bispecific aptamers efficiently degraded relevant membrane proteins of mesenchymal epithelial transition (Met) and tyrosine protein kinase-like 7(PTK-7) at nanomolar concentrations. Based on the development of SELEX technology for selecting aptamers (Sola et al., 2020), their method might allow degrading other membrane proteins. The stability and off-target effect of bispecific aptamers should be considered in further studies.

All in all, these studies established an efficient strategy for degrading the membrane proteins and extracellular proteins through lysosomal pathway, providing a potential therapeutic approach. The discovery of other recycling receptors with distinct and exclusive localization as well as their structure function studies and mechanistic investigations might be significant in propelling this field forward.

### Macroautophagy Degradation Targeting Chimeras (MADTACs): AUTACs/ATTEC

Macroautophagy is a potential degradation pathway that takes place inside eukaryotic cells, where cytosolic substrates containing proteins and large objects such as intracellular pathogens and dysfunctional organelles were engulfed and degraded by lysosomes (Khandia et al., 2019). Two independent new degrader technologies have been reported to harness the autophagy pathway.

**FIGURE 11:**
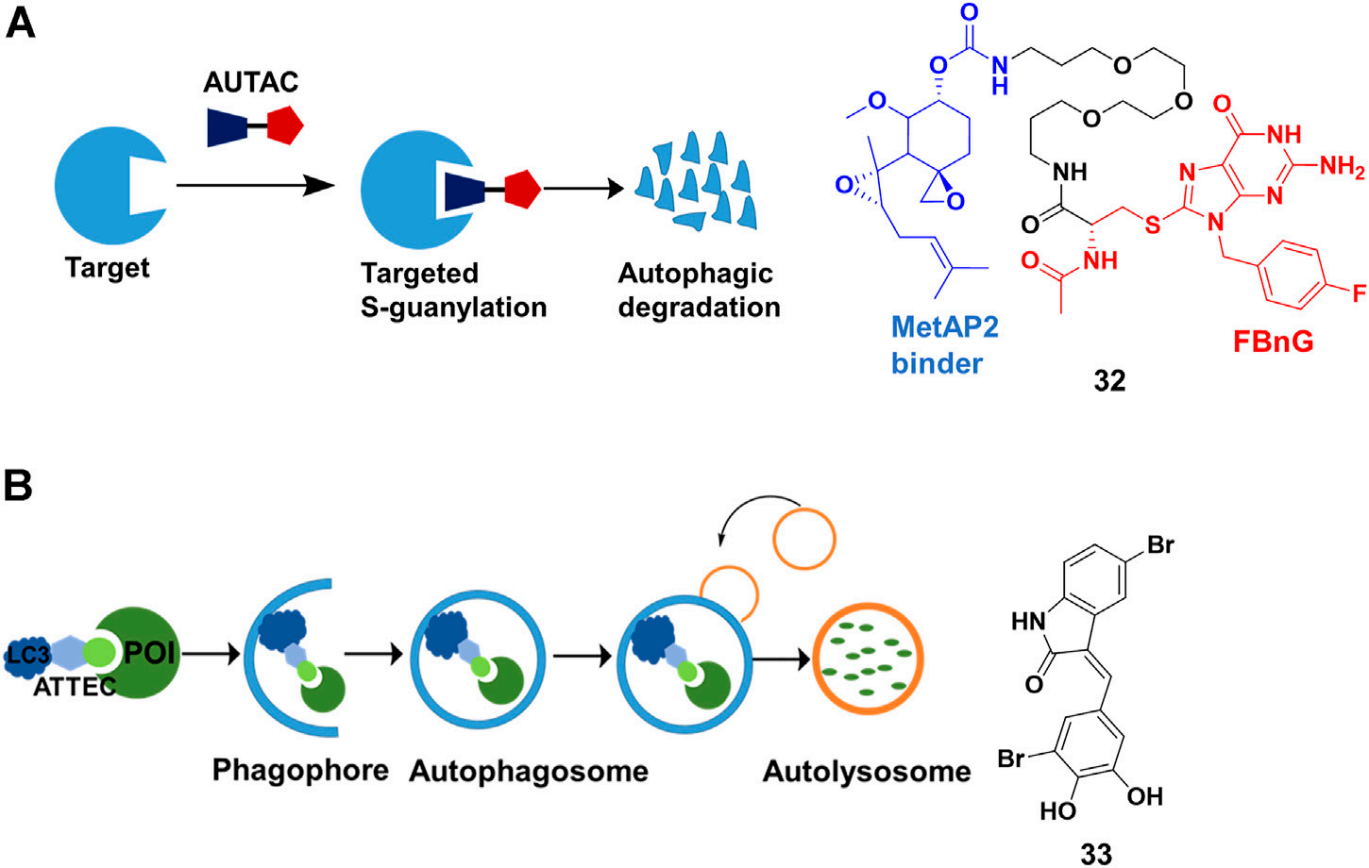
**(A)** Action mode and chemical structure of autophagy-targeting chimeras (AUTACs). **(B)** Action mode and chemical structure of autophagosome-tethering compound (ATTEC).

S- guanylation is an important posttranslational modification in xenophagy (antibacterial autophagy), which could be used to remove intracellular bacterial pathogens and activate an innate autoimmune response. Arimoto’s group (Sawa et al., 2007) demonstrated that the S-guanylation of invading group A streptococci (GAS) through the endogenous nucleotide 8-nitroguanosine 3′, 5′-cyclic monophosphate (8-nitro-cGMP) was closely correlated with subsequent K63 polyubiquitination. Then, the polyubiquitinated GAS was sequestered by GAS-containing autophagosome-like vacuoles (GcAVs), and subsequently degraded by lysosome. In a recent study (Takahashi et al., 2019), Arimoto’s group proved that the autophagy tag (Cys-S-cGMP) alone was sufficient to induce protein degradation by the autophagy pathway. Cys-S-cGMP had poor physicochemical properties, such as low membrane permeability, and might trigger activation of cGMP-dependent protein kinase G, leading to side effects. Hence, they removed cyclic phosphate moiety of the Cys-S-cGMP structure, and further optimized to generate p-fluorobenzylguanine (FBnG). Finally, they created AUTACs comprising FBnG, a linker, and a specific POI binder for degradation (Figure 11A). As expected, AUTAC **32** (Figure 11A) successfully triggered 80% degradation of methionine aminopeptidase 2 (MetAP2) at concentrations in excess of 1 μM. However, a JQ-1-based AUTAC only induced 30% degradation of BRD4 and likely only occurred during cell division, which might result from the cytoplasmic restriction of lysosomes. More importantly, they demonstrated that AUTACs could be used for the degradation of mitochondria. Further studies should focus on the exact mechanism of the degradation process that helps us to know its target spectrum and optimize structure for more potent AUTACs.

Different from AUTAC using the xenophagy pathway for degradation by tagging with FBnG, ATTEC directly engages the autophagy pathway. The autophagy pathway starts with so-called phagophore having an isolated membrane structure, derived from the lipid bilayer with lipidated light chain 3 (LC3) proteins. Subsequently, the intracellular cargoes are engulfed by phagophore, followed by isolation in autophagosomes. The loaded autophagosomes mature through fusion with the lysosomes, resulting in cargo degradation (Kundu and Thompson, 2008). Lu’s group (Li et al., 2019) presented ATTEC that interacted with both LC3 and POI, enhancing the recruitment of the POI into autophagosomes for degradation (Figure 11B). They identified **33** (Figure 11B) through small-molecule-microarray-based screening. **33** bound with both LC3 and mutant huntingtin protein (mHTT), but not with the wild-type HTT protein (wtHTT). This selectivity derived from binding the polyglutamine-repeat sites on mHTT, which was missed on wtHTT. **33** induced mHTT-selective degradation in cells at nanomolar concentrations and *in vivo*. In their recently proof-of-concept study (Fu et al., 2021), they constructed ATTEC chimeras by conjugating LC3-binding small molecules to the probes of lipid droplet (LD) through a linker. The group demonstrated that ATTEC chimeras could clear LDs, the ubiquitous lipid-storing cellular structures, through autophagic degradation and rescued LD-related phenotypes *in vitro* and *in vivo*.

The first ATTEC was similar to “molecule glues” in terms of autophagy pathway-dependent mHTT degradation. However, ATTEC chimeras were bifunctional molecules as PROTACs, and their design principle and method might be similar to those of PROTACs or other chimera degraders. Furthermore, the degradation spectrum was expanded to non-protein substrates by ATTEC chimeras.

## Degrading RNA Through Ribonuclease Targeting Chimeras (RIBOTACs)

**FIGURE 12:**
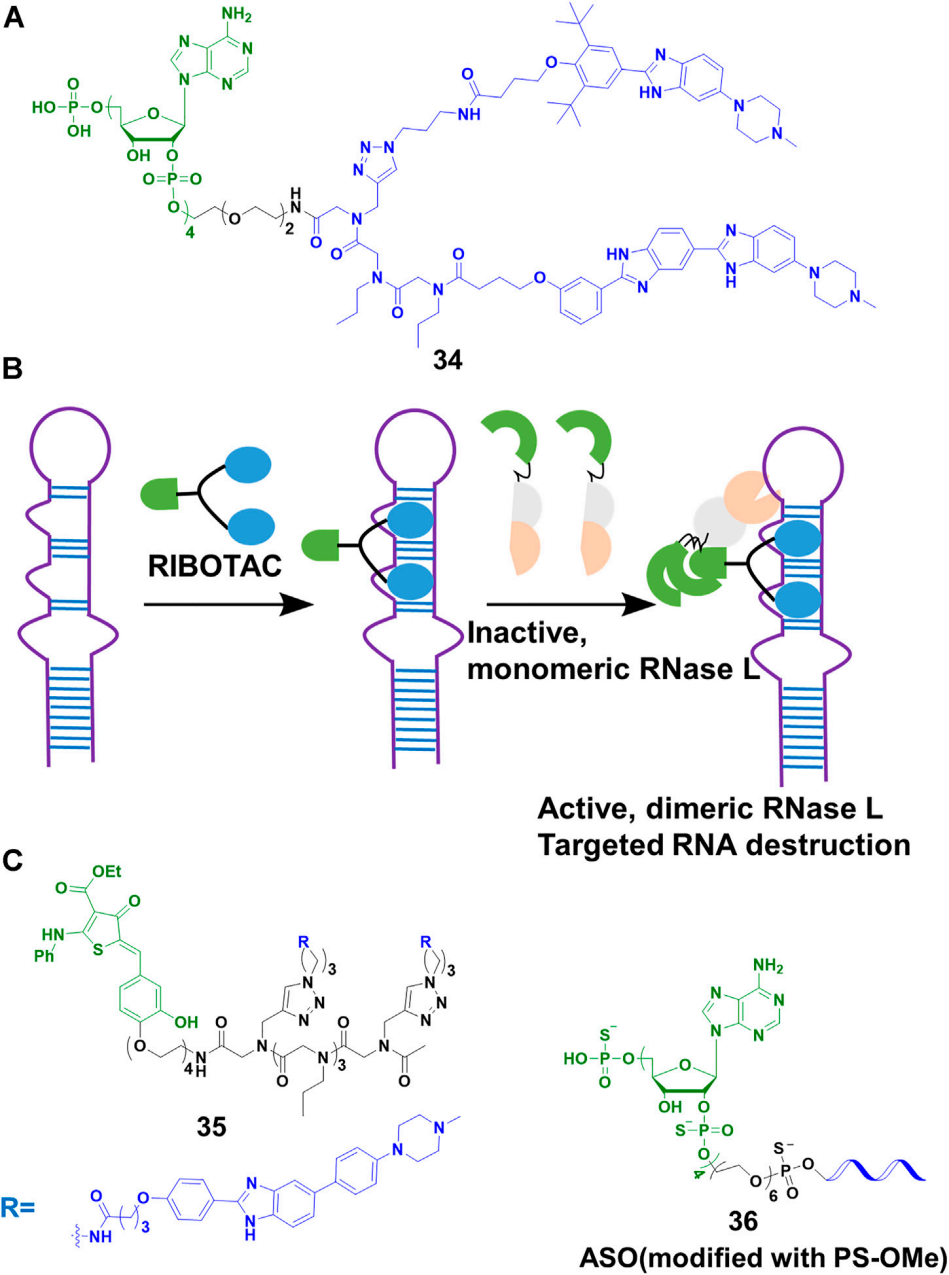
**(A)** Chemical structure of RIBOTACs. **(B)** Action mode of RIBOTACs. **(C)** Chemical structure of RIBOTACs and NATACs.

Disney’s group expanded the sphere of PROTACs from proteins to RNA. Based on their previous study on Inforna, enabling the design of small molecule targeting RNA (Velagapudi et al., 2014), Disney’s group (Costales et al., 2018) linked a small molecule targeting the primary transcript of microRNA-96 (pri-miR-96) with 2′-5′ A_4_ oligonucleotide recruiting RNase L, to construct the RIBOTACs **34** (Figure 12A). This design allowed **34** to not only recruit RNase L but also activate it only on the RNA target, and RNA target was subsequently degraded through the catalytical and substoichiometric pathway (Figure 12B). Also, **34** could increase the level of forkhead box protein O1 (FOXO1), which was controlled by microRNA-96, and result in the apoptosis of MDA-MB-231 cells, but almost no effect was found in healthy cells. In their other studies (Costales et al., 2019; Zhang P. et al., 2021), they emphasized that RIBOTACs increased the selectivity and potency comparing with simple RNA-binding compounds. For example, a small-molecule ligand that targeted precursor microRNA-210 (pre-miR-210) also bound to DNA, but the DNA binding capacity was ablated when it converted into a RIBOTAC. In a recent study (Costales et al., 2020), the group simplified the structure of the ligand of RNase L, and constructed **35** (Figure 12C) for the cleavage of precursor microRNA-21 (pre-miR-21), a key regulator of oncogenic process. Although **34** and **35** induced effective cleavage at nanomolar concentrations, **35** was more catalytic than **34**. In addition, **35** efficiently decreased pre-miR-21 level and impeded breast-to-lung cancer metastasis *in vivo*. Furthermore, RIBOTACs also were applied in mitigating amyotrophic lateral sclerosis (ALS) and frontotemporal dementia (FTD) associated pathologies *in vitro* and *in vivo* (Bush et al., 2021). Disney’s group paved the way to endow RIBOTACs with the ability to cleave the structured regions of RNA, which was not a target of sequence-based recognition technologies such as antisense oligonucleotides (Crooke et al., 2021) and CRISPR-based strategies (Pickar-Oliver and Gersbach, 2019).

So far, our lives were severely damaged by coronavirus disease 19 (COVID-19). Many scientific communities tried to pursue multiple strategies to address COVID-19. As the process of RNA virus replication and RNase L were located in the cytoplasm, targeting the degradation of severe acute respiratory syndrome coronavirus 2 (SARS-Cov-2) that caused COVID-19, was seen as a potential treatment. Disney’s group (Haniff et al., 2020) designed RIBOTACs to target the SRAS-CoV-2 frameshifting element, controlling the translation of polyproteins pp1a and pp1ab which played a vital role in viral replication and pathogenesis. Their results showed that SARS-CoV-2 frameshifting element was degraded at micromolar in cells. Furthermore, our team (Su et al., 2021) developed a nucleic acid targeting chimeras (NATACs) through connecting an antisense oligonucleotide (ASO) with RNase L binder 2′-5′ A_4_. The complementary oligonucleotide sequence was applied to search and bind to the target viral RNA, while the 2′-5′ A_4_ recruited RNase L. The NATACs based **36** (Figure 12C) successfully degraded the spike RNA of SARS-CoV-2. In our pseudovirus infection models, the spike RNA and N501Y and/or ΔH69/ΔV70 mutants of SARS-CoV-2 were decreased efficiently (80 nM). Comparing with ASO without 2′-5′ A_4_ oligonucleotide, **36** enhanced more than 2-fold degradation efficiency. Although the two studies were not applied in live viruses, they proved a new strategy to combat COVID-19 and other viruses.

## Conclusion

To sum up, various degrader technologies had been developed to degrade proteins or RNAs via different pathways in the last 3 years. Firstly, most research efforts highlighted here demonstrated that modified PROTACs was a useful strategy for improving degradation specificity of PROTACs such as light-controllable PROTACs and antibody/aptamer/folate-PROTACs conjugates. Secondly, some atypical PROTACs were introduced such as covalent PROTACs, trivalent PROTACs, and nucleic acid-based PROTACs, these novel PROTACs expanded the degradation spectrum. Thirdly, some emerging degraders hijacked the lysosomal degradation pathway for POI degradation, such as LYTACs, AUTACs and ATTEC. Although all of these protein degraders achieve degradation via lysosome-based pathway, they takes advantage of different degradation mechanism. LYTACs used a glycan tag to mark the extracellular targets for degradation, whereas AUTACs contained an S-guanylation tag to induce POI degradation through selective autophagy. Furthermore, ATTEC exploited lipidated LC3 to straightforwardly tether targets to growing autophagosomes, facilitating their selective removal via autophagy. Beyond these protein degraders, RIBOTACs and NATACs had been used to target RNA implicated in cancer and viruses, expanding the target spectrum of bifunctional chimera molecules to RNA. At present, these degrader technologies are merely at earlier evolutionary stage, and have key concerns needed to be addressed. Most of modified PROTACs need complicated synthesis skills and increase the molecular weight, leading possibly to the poor permeability such as trivalent PROTACs and nucleic acid-based PROTACs. In addition, current light-controllable PROTACs need UV/visible light to active, but the use of these wavelengths to activate is incompatible with clinical applications that require deep tissue penetration. Also, efforts should be made to charactering degradation mechanism especially for AUTACs. Given these significant studies, classical PROTACs filed has expanded the possibility of how intracellular protein can be modulated. It also expands the degradation spectrum of PROTACs to extracellular protein and RNA.
